# Role of Calpain in Apoptosis

**Published:** 2011-08-24

**Authors:** Hamid Reza Momeni

**Affiliations:** Biology Department, Faculty of Science, Arak University, Arak, Iran

**Keywords:** Apoptosis, Calpain, Calpain Substrates

## Abstract

Apoptosis, a form of programmed cell death that occurs under physiological as well as pathological conditions, is characterized by morphological and biochemical features. While the importance of caspases in apoptosis is established, several noncaspase proteases (Ca^2+^-dependent proteases) such as calpain may play a role in the execution of apoptosis. The calpain family consists of two major isoforms, calpain I and calpain II which require µM and mM Ca^2+^ concentrations to initiate their activity. An increase in intracellular Ca^2+^ level is thought to trigger a cascade of biochemical processes including calpain activation. Once activated, calpains degrade membrane, cytoplasmic and nuclear substrates, leading to the breakdown of cellular architecture and finally apoptosis. The activation of calpain has been implicated in neuronal apoptosis following spinal cord injuries and neurodegenerative diseases. This review focuses on calpain with an emphasis on its key role in the proteolysis of cellular protein substrates following apoptosis.

## Introduction

### Apoptosis

The Greek term "apoptosis" was first used by Kerr et al. in 1972. Originally it referred to the "falling off" or "dropping off" of petals from flowers or leaves from trees (1). Apoptosis is considered to be an endogenous, active cellular process by which an external signal activates metabolic pathways resulting in cell death (2). This form of cell death appears to be a morphologically and biochemically distinct form of eukaryotic cell death that can be triggered by a variety of physiological and pathological conditions (3).

Apoptosis is an essential mechanism for eliminating unwanted neuronal cells during the development and homeostasis of multicellular organisms. During metamorphosis, cell death is rapidly apparent in both insects and amphibians where the larval tissues must be replaced by those of the adult (4). In mammals, apoptosis is conspicuous from the very beginning of development. In animals, during the development of the nervous system, motor neurons are generated in larger numbers than needed. For instance, in the lumbar spinal cord of the developing rat about 6000 motor neurons are present at embryonic day 14. These neurons grow out axons with the intention of contacting their target tissue, the skeletal muscles. However, about 50% of the motor neurons do not successfully establish target contact and are lost during the critical period from day 14 to postnatal day 3 (5). This process is called physiological motor neuron death. Apoptosis also occurs during development of the gut, limb buds, cartilage and bones (4). Furthermore, apoptosis is critical for the maintenance of normal homeostasis. For instance, in adult mammals apoptosis occurs continually both in slowly proliferating cell populations, such as the epithelium of the liver, prostate, and adrenal cortex, and in rapidly proliferating populations, such as the epithelium which lines the intestinal crypts and differentiating spermatogonia (6).

Apoptosis is the main cause of cell death observed in pathological conditions in the central nervous system. For instance, apoptosis has been described in neurons and glial cells during spinal cord injury as well as in many neurodegenerative diseases such as amyotrophic lateral sclerosis, Parkinson’s and Alzheimer’s disease (7).

### Morphological and biochemical characterization of apoptosis

Classical apoptotic cell death can be defined by both morphological and biochemical characteristics that distinguish it from other forms of cell death.

### Morphological features

**Fig 1:**
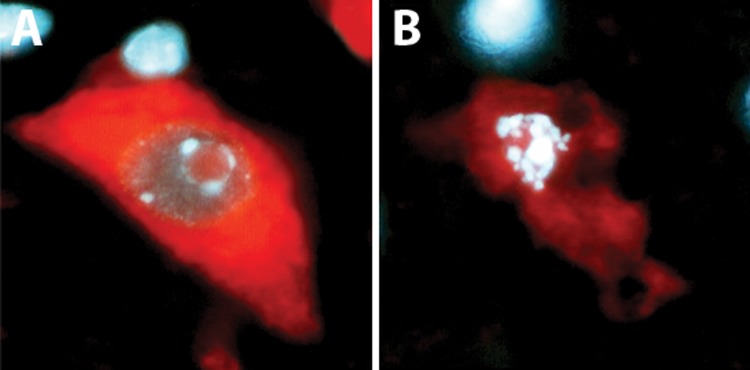
Apoptosis in a motor neuron of the adult mouse spinal cord in a slice culture. A combination of propidium iodide (red) and Hoechst (blue) stained motor neurons. A. Motor neuron from freshly prepared slices (0 hour). B. Apoptotic motor neuron from slice cultured for 6 hours shows clear cell shrinkage as well as nuclear and chromatin condensation

One of the earliest events during apoptosis is cell dehydration where the loss of intracellular water leads to cellular shrinkage. Another change, perhaps the most characteristic feature of apoptosis, is nuclear and chromatin condensation (Fig 1).

These events are followed by nuclear fragmentation. The nuclear fragments, together with the constituents of the cytoplasm (including organelles) are then packaged and enveloped by fragments of the plasma membrane to form apoptotic bodies. When apoptosis occurs in vivo, apoptotic bodies are phagocytized by neighboring cells in the absence of an inflammatory reaction in the tissue (8). Another feature of apoptosis, at least during the initial phase, is preservation of the structural integrity of plasma membrane and cellular organelles, including mitochondria and lysosomes, although the mitochondrial transmembrane potential is markedly decreased (8).

### Biochemical feature

**Fig 2:**
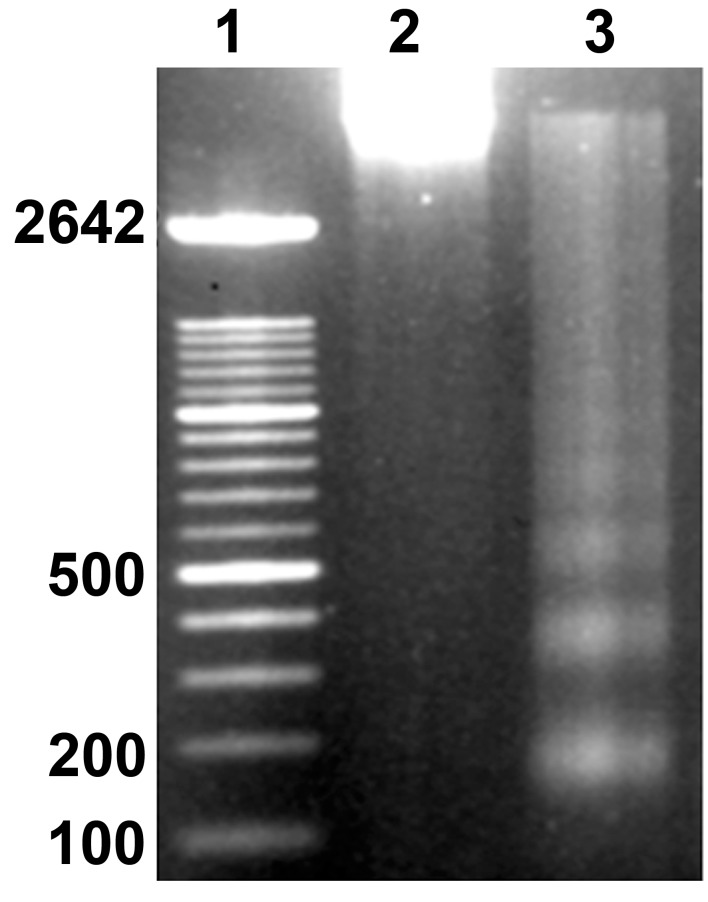
Agarose gel electrophoresis of DNA isolated from adult spinal cord slices in culture. Lane 1: Markers presented as base pairs. Lane 2: High molecular weight DNA from fresh slices (0 hour). Lane 3: Nucleosomal DNA fragmentation in slices cultured for 24 hours.

The formation of distinct DNA fragments of nucleosomal size (approximately 180 bp) is a biochemical hallmark of apoptosis in most cell lines and tissues (9). These fragments generate a ladder of DNA when analyzed by agarose gel electrophoresis (Fig 2). However, there are several apoptotic models in which there is no nucleosomal DNA cleavage (10).

### Noncaspase protease, calpain

While the importance of caspases (Ca^2+^-independent proteases) in apoptosis have been clearly established, studies have indicated that several other types of noncaspase proteases may also play a role in the execution of apoptosis. Noncaspase proteases most closely linked to apoptosis are calpains, cathepsins, granzymes and the proteasome (11).

Here, we focus on the calpain family, which is a family of Ca^2+^-activated neutral cysteine, nonlysosomal endoproteases found in all mammalian cells (12). The calpains can be broadly classified into two major groups based on their tissue distribution; calpains that are tissue-specific and calpains that are ubiquitously expressed. The tissuespecific group includes skeletal muscle-specific (calpain-3) and stomach-specific (calpain-9) calpains. The best-characterized ubiquitous calpains in mammals are µ-calpain (calpain I) and m-calpain (calpain II). They can be distinguished by their *in vitro* requirement for different levels of Ca^2+^ for activation, 2-80 µ M for calpain I and 0.2- 0.8 mM of Ca^2+^ for calpain II (13).

### Structure and biochemical properties of calpain

**Fig 3:**
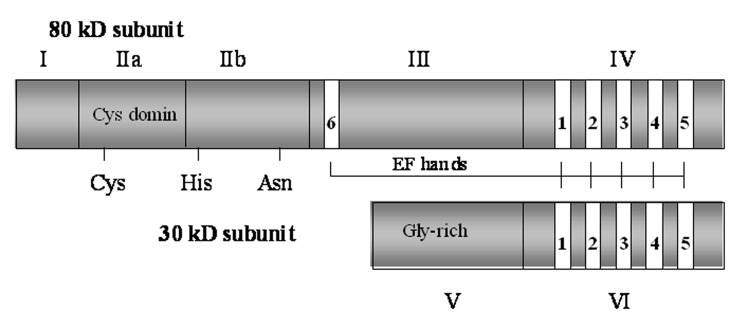
Domain structure of calpain subunits. The large subunit and small subunit contain four and two domains, respectively. E-helix-loop-F helix (EF hands) located in domains III, IV and VI are calmodulin-like Ca^2+^ binding sites (Modified from reference 13).

Both calpain I and calpain II are heterodimers composed of a large (80 kD) catalytic subunit and a small (30 kD) regulatory subunit (Fig 3). The 80 kD subunit can be divided into four domains (I, II, III and IV) and the 30 kD subunit into two domains (V and VI). Domain I is the N-terminal region of the catalytic subunit and contains the site where autolytic cleavage occurs prior to or parallel to the proteolysis of substrates (Fig 3).

Domain II is composed of two subdomains (IIa and IIb). The active site Cys on IIa interacts with both the substrate and the inhibitory region of calpastatin. The exact function of domain III is unknown. Domain IV is the C-terminal end of the large subunit. It is structurally similar to calmodulin with five Ca^2+^ binding sites, which are E-helix-loop-F-helix motifs (EF hands). Another EF hand is present at the beginning of domain III. Domain V, the N-terminal region of the regulatory subunit, is hydrophobic because of glycine clustering and may function as a membrane anchor. Domain VI, the C-terminal end of the small subunit, is a Ca^2+^ binding region similar to that of the large subunit (13).

### Activation mechanism of calpain

The activation of calpain has been implicated in neuronal death following spinal cord injury (14), multiple sclerosis, cataract, stroke (neuronal ischemia) (15) as well as in the central nervous system and neurodegenerative diseases such as Alzheimer's (16), Parkinson's (17) and amyotrophic lateral sclerosis (18). Calpain is reported to be responsible for the apoptosis of glial cells (19, 20). Recently, we have also shown calpain activation in the motor neurons of adult mouse spinal cord slices which suggests a possible role of calpain in the apoptosis of adult motor neurons (21).

Calpain exists as an inactive proenzyme in the cytosol, where the normal range of intracellular free Ca^2+^ concentration is 50-100 nM in resting cells (22). An increase in the intracellular free Ca^2+^ concentration triggers the activation of calpain (14). Under physiological conditions the activation of calpain is likely to be stimulated by transient localized increases in cytosolic Ca^2+^ concentration and it is tightly regulated by the presence of an endogenous inhibitor protein, calpastatin. Under pathological situations the regulation of calpain activity may be perturbed due to elevations in intracellular free Ca^2+^ (23).

**Fig 4:**
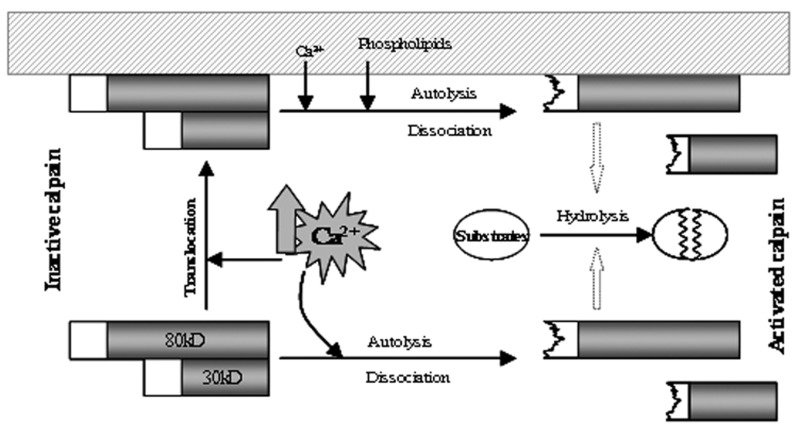
Schematic autolysis/dissociation mechanism for the activation of calpain.

The following mechanisms have been proposed to account for the activation of calpain in vivo (Fig 4). The first mechanism proposes that an increase in intracellular free Ca^2+^ concentration triggers an autolysis of N-terminal propeptide proteins of both subunits, which results in a conformational change in the molecule and the separation of truncated subunits, leading to enzyme activation (13). Activated calpain then cleaves its substrate proteins. Thus, subunit autolysis seems to be an important early event for dissociation and calpain activation (24). The second mechanism proposes that a high concentration of intracellular Ca^2+^ triggers translocation of inactive calpain from the cytosol to the membrane. At the membrane, calpain is activated in the presence of Ca^2+^ and membrane effectors such as phospholipids (24). The autocatalytic hydrolysis of domain I occurs during activation, resulting in the dissociation of 30 kD from 80 kD. Activated calpain hydrolyzes substrate proteins either in the membrane or cytosol after its release from the membranes (25). Translocation to the membrane might be one of the important steps necessary to separate calpain from its endogenous inhibitor, calpastatin (24).

A third alternative is the phosphorylation of calpain, for instance, by protein kinase A at Ser-369 in domain III. This might be another important mechanism for the activity regulation of calpain (25).

### The role of calpastatin in controlling calpain activation

**Fig 5:**
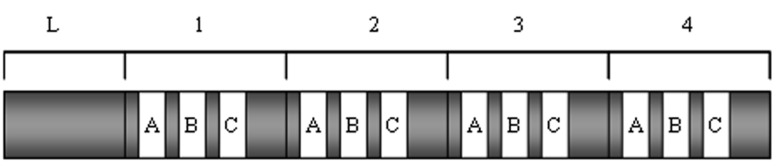
Domain structure of calpastatin (Modified from reference 13).

In addition to the mechanisms mentioned above, the activity of calpain is regulated by calpastatin. Calpastatin, a ubiquitously-expressed protein, is a specific endogenous inhibitor for the regulation of proteolytic activity of ubiquitous calpains in mammalian cells (26). Calpastatin is very specific for µ- and m-calpain, and co-exists with calpain in the cytosol (27) and membrane (28). Calpastatin (110 kD) is comprised of an N-terminal domain L and four repeated domains (Fig 5), each of which is able to independently inhibit calpain. One molecule of calpastatin can therefore inhibit four molecules of calpain (13).

Molecular interaction of calpastatin with calpain prevents the production of the autolytic form thus inhibiting calpain’s catalytic activity (29).

Calpastatin is normally present in large excess in the cell as compared with calpain. In vitro, it inhibits both the native and auto-proteolysed form of the proteinase (27). It has been proposed that the regulation of calpain activity by calpastatin decreases following the digestion of calpastatin by calpain. This dysregulation can occur due to an increase in the ratio of calpain to calpastatin and substantially increases in spinal cord injury and other neurophatophysiological conditions. Following spinal cord injury, this increased ratio is thought to degrade calpastatin into small fragments, therefore losing its regulatory influence on calpain (13).

Averna et al. (27) have suggested that following an increase in intracellular Ca^2+^, calpastatin is released from its association with calpain and results in calpain activation.

Finally, the phosphorylation of calpastatin by various protein kinases, such as protein kinase C and protein kinase A, has also been suggested as a mechanism for altering inhibitory specificity in rat skeletal muscle and inhibitory efficiency in the rat brain (27).

### Calpain substrates

A large variety of proteins are calpain substrates. They include cytoskeletal proteins such as α-fodrin and neurofilaments, membrane proteins such as ion channels, growth factor receptors, adhesion molecules as well as enzymes (30) and protein constituents of myelin (myelin basic protein) (31). Several calpain substrates are also located in the nucleus such as the nucleoskeletal proteins lamin A and B (32). Interestingly, calpastatin can also be a substrate for calpain. In this context, Pontremoli et al. (33) have shown the degradation of calpastatin by calpain, as a suicide substrate, due to an increase in the calpain/calpastatin ratio.

α-fodrin is the best calpain substrate. Saido and coworkers (34), in a postischemic hippocampus, have shown that activated calpain degraded the 230-kD α-fodrin into a 150-kD fragment. They reported that the appearance of 150-kD calpain-cleaved α-fodrin fragment could be inhibited by leupeptin, a calpain inhibitor.

### Calpain activity assay, substrate hydrolysis

Cellular and synthetic calpain substrates can be used as indicators for measuring calpain activity in cells and tissues. α-fodrin, which is one of best calpain substrates has been used for calpain activity and apoptosis during spinal cord injury (35). In accordance with this, we have used a Western blot analysis of the production of 150- kD calpain-cleaved α-fodrin fragment to assess calpain activity in motor neurons of spinal cord slices (21).

A synthetic and cell-permeable fluorogenic calpain substrate, t-butoxycarbonyl-Leu-Met-7-amino-4 -chloromethylcoumarin (Boc-Leu-Met-CMAC), is another specific calpain substrate that directly assesses calpain activity in living cells (36,37) and tissues (38). This substrate is non-fluorescent, but after diffusion into the cells it becomes enzymatically conjugated to protein thiol groups. Subsequent proteolytic hydrolysis by calpain unquenches the fluorescent, membrane-impermeable MAC-thiol moiety within the cell (37). The fluorogenic calpain substrate has been shown to be specific for calpain activity and widely used in a variety of living cells in vitro (36, 39-41) and tissues. With this substrate we (21) demonstrated calpain activity in apoptotic motor neurons in cultured spinal cord slices.

### Calpain in apoptosis, substrate hydrolysis

**Fig 6:**
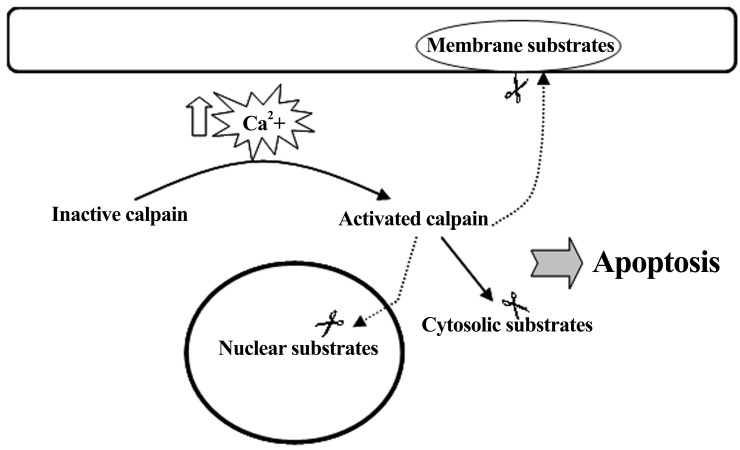
Calpain is activated by intracellular Ca^2+^ overload. Once activated, calpain hydrolyses its substrates in the cytosol, nucleus and membrane, resulting in apoptosis.

Calpain activation in apoptosis was first demonstrated in thymocytes, as measured by calpain autolysis (42). Calpain has also been implicated in neuronal apoptosis during spinal cord injury (14). Calpain's role in apoptosis has been confirmed by the inhibition of apoptosis by calpain inhibitors in a variety of neurons; including motor neurons from adult mouse spinal cord slices (43,44), chicken spinal motor neurons, dorsal root ganglion neurons (45) and hippocampal neurons (46). The involvement of calpain in neuronal apoptosis is further suggested by additional evidence which shows that activated calpain mediates the degradation of many cytoskeletal and membrane proteins (31) as well as various structural proteins in the nuclear matrix, such as lamins (32) (Fig 6). These proteins are involved in maintaining neuronal structural integrity which is essential for normal cellular function and survival, and their degradation leads to apoptosis (47).

### Calpain inhibitors

To date, a variety of calpain inhibitors have been synthesized. Leupeptin, for instance, improves motor neurons survival in rat embryos (48). The more specific calpain inhibitors [calpain inhibitor VI, SJA6017; and calpain inhibitor XI, Zl- Abu-CONH (CH2)_3_-morpholine] have been shown to protect both retinal (49) and cortical neurons (50), respectively, against ischemiainduced damage.

We have also shown that both inhibitors had a protective effect on the apoptosis of motor neurons in spinal cord slices (43, 44). Furthermore, evidence also shows that both the calpain inhibitor VI and XI were able to block calpain activity in mouse retinal photoreceptors (38). Using spinal cord slice culture, we also demonstrated that calpain inhibitor VI could inhibit calpain activity in motor neurons (21).

Although ethyleneglycol-bis (b- aminoethyl ether) N, N, N', N'-tetraacetic acid (EGTA) is not a calpain inhibitor, it mimics the effects of calpain inhibitors by chelating extracellular Ca^2+^. EGTA has been shown to inhibit calpain activation in motor neurons of spinal cord slices (21) and the cultures of rat oligodendrocytes (20). The most specific calpain inhibitor is the protein calpastatin. It directly binds to the Ca^2+^ binding domains of both the large and small subunits of calpain (12). The inhibitor has a high molecular mass and is therefore membrane impermeable (13), limiting its use as a pharmacological tool.

### Calpain and neurodegenerative disorders

Disorders such as cerebral ischemia, Alzheimer's, Parkinson's, and Huntingtons disease, amyotrophic lateral sclerosis and multiple sclerosis are neurodegenerative diseases in which neurons in the central nervous system die. Increased intracellular Ca^2+^ concentration and calpain activation are thought to be responsible for the induction of neuronal death in these diseases (51).

The initial pathology of cerebral ischemia is caused by energy depletion to affected brain regions (52). Uncontrolled release of glutamate (53) and impairment in its reuptake results in calcium influx through the glutamate receptors and ultimately excitotoxicity (54).

Several lines of evidence have demonstrated that increased levels of soluble amyloid β-protein (Aβ) are the primary cause of neuronal pathology in Alzheimer's (55). Higher concentrations of soluble Aβ result in higher intracellular calcium concentration via the Nmethyl- D-asparate (NMDA) receptor (56).

The pathophysiology underlying the degeneration of substantia nigra dopaminergic neurons in Parkinson's disease is proposed to be due to mitochondrial dysfunction, leading to increased free radical production and higher intracellular calcium concentrations (57).

One hypothesis concerning the mechanism of a mutation in the Huntington protein which results in neuronal death in Huntington's disease is that the mutant Huntington protein induces mitochondrial defects through the inhibition of mitochondrial complex II—succinate dehydrogenase, (58) leading to aberrant calcium homeostasis (59).

The pathogenesis of amyotrophic lateral sclerosis, a progressive neurodegenerative disorder leading to motor neuron loss, axonal degeneration, muscular atrophy and death (60) is thought to be due to excess extracellular glutamate (61) and excitotoxicity.

Loss of myelin proteins in multiple sclerosis occurs by protease-mediated breakdown. Since all major myelin proteins, including myelin basic protein and axonal neurofilament protein are the substrates of calpain (62), myelin loss might be correlated with calpain activity.

In all neurodegenerative disorders in which calcium homeostasis is altered, calcium dysregulation leads to the pathologic activation of calpain (63). Calpain activation then induces cleavage of a number of proteins involved in the homeostatic control of intracellular calcium. Calpain cleavage of the plasma membrane calcium ATPase (64), sodium-calcium exchanger (65), L-type calcium channel (66), ryanodine receptor (67), sarcoplasmic/endoplasmic reticulum calcium ATPase (68) and inositol 1, 4, 5 triphosphate receptor (69) are the result of elevated intracellular calcium levels.

Calpain also cleaves key cytosolic enzymes involved in calcium homeostasis such as Ca^2+^/calmodulin- dependent protein kinase type IV (70).

## Conclusion

Calpain is calcium-activated protease which exists as an inactive proenzyme in the cytosol. When intracellular calcium level is overloaded, it triggers to convert the proenzyme to its active form. Activated calpain then cleaves cytoplasmic and nuclear substrates, leading to apoptosis.
